# Safety and efficacy of a single intra-articular injection of hyaluronic acid in osteoarthritis of the hip: a case series of 87 patients

**DOI:** 10.1186/s12891-021-04672-0

**Published:** 2021-09-16

**Authors:** David M. Long, Jane Fitzpatrick

**Affiliations:** 1 Olympic Park Sports Medicine Centre, 60 Olympic Blvd, Melbourne, 3004 Australia; 2 School of Medicine, Deakin University, Little Malop St, Geelong, Victoria 3220 Australia; 3 Centre for Health and Exercise Sports Medicine, Faculty of Medicine, Dentistry and Health Science, University of Melbourne, Level 7, Alan Gilbert Building, 161 Barry Street, Parkville, Victoria 3010 Australia; 4 Joint Health Institute, Malvern, Victoria 3144 Australia

**Keywords:** Durolane, Modified Harris hip score, Hip joint, NASHA

## Abstract

**Background:**

Osteoarthritis (OA) is the most prevalent form of joint disease and commonly affects the hip. Hip OA is associated with a high socioeconomic burden. Intra-articular hyaluronic acid (HA) injection may be of benefit but quality evidence for HA use in hip OA is lacking. The purpose of this study was to assess the safety and efficacy of ultrasound guided injection of a high molecular weight, non-animal derived, stabilised HA (NASHA) in patients with mild to moderate hip OA.

**Methods:**

This single site study is an analysis of prospectively collected outcome data for 87 consecutive patients over a 2-year period who received a single HA (Durolane) injection for symptomatic hip OA. Inclusion criteria were male or female patients over 18-years of age with mild to moderate hip OA on x-ray. Patients with severe hip OA were excluded. The primary outcome measure was a modified Harris Hip Score (mHHS) questionnaire at baseline and 6-weeks with a minimal clinically important difference (MCID) of 10 points. All adverse events were recorded and assessed.

**Results:**

Data from 87 patients, 49 women and 38 men with mean age of 54 (SD = 10.8) were analysed. At baseline, mean mHHS was 58.47 (SD 14.31). At the 6 week follow up, mean mHHS improved to 71.30 (SD 16.46), a difference of 12.83 (*p* < 0.01). This was greater than the MCID of 10. No significant adverse events were encountered. Five patients reported short-lived injection site pain.

**Conclusion:**

A single injection of HA (NASHA) in the setting of hip joint OA was both safe and efficacious in this 87 patient cohort. Improvement in pain and function as measured with mHHS was statistically significant and reached the MCID of 10.

**Trial registration:**

The study was retrospectively registered on the 1st of February 2021 in the Australian New Zealand Clinical Trials Registry with registry number ACTRN12621000098831. All research was performed in accordance with the Declaration of Helsinki.

## Introduction

Osteoarthritis (OA) is the most prevalent form of joint disease and a significant cause of morbidity worldwide [23, 37] It is characterised by progressive degradation of cartilage, remodelling of bone, pain and progressive loss of range of motion and function [40]. In the 2016 WHO Global Burden of Disease Study, OA was the thirteenth ranked cause of years lived with disability in the world rising from sixteenth in 2000 [11, 38, 37].

The hip joint is the second most commonly affected joint in which OA occurs, affecting approximately 3–11% of people over the age of 35, rising with age such that over the age of 60, prevalence is in the order of 10–30% [11, 41].

The socioeconomic costs of hip OA have increased by 80% in the past ten years [37, 41]. In Australia alone the estimated total cost of OA is $5.5 billion [15]. The majority of these costs are due to joint replacement surgery for end stage disease and premature exit from the workplace [15]. In 2018 49,972 hip replacements were performed, a 125% increase since 2003 [3]. Of these, 36% were done in patients under the age of 65, with a 2% increase in the number of younger patients having hip replacements since 2003 [3].

There is no cure for OA and to date, most treatments have focused on alleviating pain and preventing functional decline. Whilst there is good evidence for weight loss and exercise in the management of knee OA, exercise only has a small to moderate effect in the hip [17]. The use of paracetamol and non-steroidal anti-inflammatory drugs is common but the effect sizes of these are small and the use of these in the long term and at higher doses can lead to gastrointestinal and hepatic side effects. The prescription of opioids for OA has increased in recent years despite guidelines recommending against their use [5, 6, 36]. The use of intra-articular steroids has been shown to be effective only in the short term and is associated with a long-term risk of an increased rate of progression to OA in the knee [28]. It may thus potentially hasten joint replacement.

Total hip joint replacement does provide relief and restoration of function in the vast majority of patients with end-stage disease. However, the procedure is associated with not insignificant operative risk and post-operative systemic and local complications including venous thromboembolism (0.5%) along with a 1–5% risk of complications pertaining to the prosthesis such as infection or dislocation [30, 20]. There is also an approximate 10% cumulative revision rate for primary total hip joint replacement in OA at 15 years [3]. With these risks and costs in mind, and in the context of an aging population, continuing increases in life expectancy and many younger patients becoming affected by OA there is an urgent need to find alternative treatments for symptomatic relief and improved function.

Intra-articular injection of hyaluronic acid (HA), also referred to as viscosupplementation, is widely used for the symptomatic management of knee OA. The previously held view that OA is a “wear and tear” condition has been supplanted by an increasing understanding of the complex molecular biology of OA which includes the interactions between a number of inflammatory cytokines and matrix metalloproteinases which can lead to an imbalance between cartilage matrix synthesis and degradation [1]. Hyaluronic acid is a critical constituent of healthy synovial fluid responsible for it’s viscoelastic properties [29, 35]. It has direct effects on pain via mechanosensitive, pain transducing ion channels and sensitised nociceptor terminals in addition to chondroprotective effects through decreased inflammatory cytokines, and enhanced proteoglycan and glycosaminoglycan synthesis [1, 19, 27, 32, 33]. The concentration and molecular weight of HA in patients with osteoarthritic joints is significantly lower than in healthy subjects [29].

Several studies have evaluated the use of HA in OA of the hip [7, 9, 10, 12, 14, 16, 19]. A recent meta-analysis concluded that intra-articular HA in hip OA can significantly reduce pain and improve functional recovery when compared with the condition before treatment [10]. However, this analysis concluded that there was no significant difference between HA and saline or other treatments [10]. Of note, this study did not differentiate between different HA preparations. Hyaluronic acid preparations differ in the source (animal or bacterial fermentation), volume, molecular weight of the HA, cross-linking agent, half-life or residence time and concentration [7]. These differences can shorten residence time of HA in the joint, alter efficacy and the side effect profile. Two recent reviews have concluded that the optimal HA for use in OA should be derived from biological fermentation with a molecular weight of > 3000 kilodaltons and a longer residence time [2, 24]. The recent consensus statement of the Arthroscopy Association of Canada regarding intra-articular injection in knee OA further supports this, concluding that high molecular weight and highly cross linked HA are superior to low molecular weight and non-cross linked HA [4]. There are also differences in outcomes between animal and non-animal derived HA - animal derived HA can also be associated with avian allergy, causing local or systemic allergic response [31]. In line with these recommendations, a commercially available HA; non-animal derived, stabilised HA (NASHA) with molecular weight of > 3000 kilodaltons (Durolane; Bioventus Global LL, Netherlands) was selected for this study.

The purpose of this study was to assess the safety and efficacy of HA (Durolane) in reducing pain and improving function in hip OA in the clinical setting.

## Methods

### Trial design

This single site study is a prospective analysis of outcome data for 87 consecutive patients over a 2-year period who received a single HA (Durolane) injection for symptomatic hip OA. The study was approved by the Human Research Ethics Committee at the University of Melbourne (1,853,383.1). This was an open-labelled single arm trial with participants, clinical investigator (treating physician), and investigators examining the data were not blinded to the treatment allocation. Informed consent was obtained.

### Participant selection

Patients were eligible for inclusion if they were > 18 years old, presenting during the 2 year study period with symptomatic hip joint OA (Grade 2–3 Kellgren-Lawrence as determined by the reporting radiologist). Patients presenting with severe (Kellgren-Lawrence grade 4) OA, inflammatory arthropathy or avascular necrosis were excluded. Patients who had not undertaken a trial of adequate conservative management (physiotherapy) were excluded. Past arthroscopic surgery or trauma were not exclusion criteria. There was no maximum age limit applied to the inclusion criteria. There were no specific inclusion or exclusion criteria in regards to race or gender.

### Intervention

All patients received a single injection of HA (Durolane; 3 ml preparation) in accordance with the manufacturer’s guidelines. This was performed with the patient supine using aseptic technique under ultrasound guidance. A sterile 21 g spinal needle was introduced via an anterolateral approach under direct in-plane ultrasound guidance with infiltration of 2–3 mls of lignocaine 2% (Xylocaine, Astra Zeneca, UK) sub-cuticularly and on the hip capsule followed by injection of Durolane 3mls intraarticularly. Standard post procedure advice was provided. Strenuous activity was discouraged for 48 h after the injection after which patients were advised to continue their current physiotherapy or exercise regimen. Notifications of any complications or adverse outcomes were also sought and recorded. Each patient was provided with written and verbal information regarding the procedure and written consent was obtained.

### Outcome measures

The modified Harris Hip Score (mHHS) was used as the primary outcome measure. This was completed at baseline and 6 weeks post injection. The mHHS is a validated hip score which was derived from the Harris Hip Score after removing the physician reported range of movement assessment but retaining the pain and functional scores [13, 26]. These two scores have been shown to be comparable [13].

### Statistical analysis

Data was collected prospectively in the medical record and transferred to a Microsoft Excel® spreadsheet. Each patient record was assigned a unique study identification number. The results were entered on a locked Excel spreadsheet (Microsoft), coded, and analysed. Statistical analysis utilising a two sample t-test with equal variances was conducted using STATA version 15 (StataCorp). Treatment comparisons were based on the change in mHHS from baseline to 6 weeks, using the mean change with *p*-values < 0.05 required for statistical significance and 95% confidence intervals and a MCID of 10 points.

## Results

### Participants

**Fig. 1:**
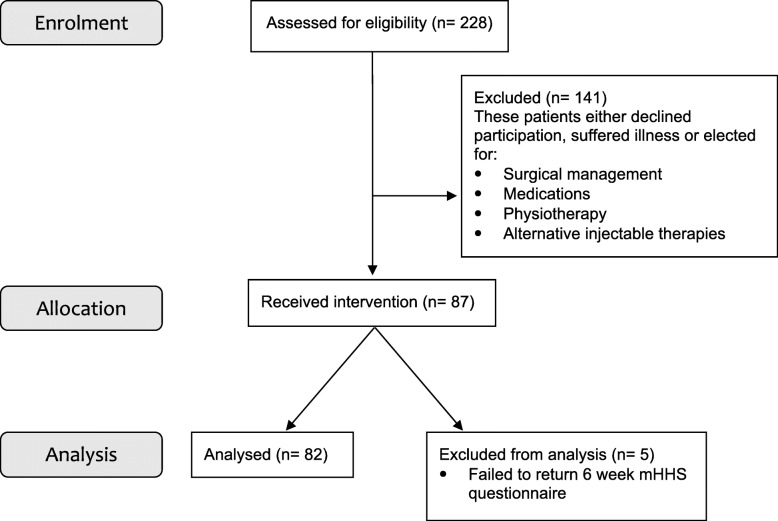
CONSORT Flow Diagram

During the recruitment period from January 2016 until December 2017, 228 patients with hip OA were screened. One hundred forty-one patients were excluded for the following reasons: declined to participate, no adequate trial of physiotherapy or selection other treatments such as medications, other injections or surgery. Data was assessed from 87 patients. Table 1 shows the demographic data for the participants. There were 49 women and 38 men aged between 26 and 82 (mean age 54; SD = 10.8). Five patients failed to complete scores at 6 weeks leaving 82 patients for analysis. The process of selection and exclusion is outlined in the CORSORT flow diagram (Fig. 1).

**Table 1:** Demographic data

Parameter	Mean (SD)
Age (years)	54 (10.8)
Parameter	N (%)
Sex	
Males	38 (43.7%)
Females	49 (56.3%)

Legend: *SD* Standard deviation, *N* Number

**Fig. 2:**
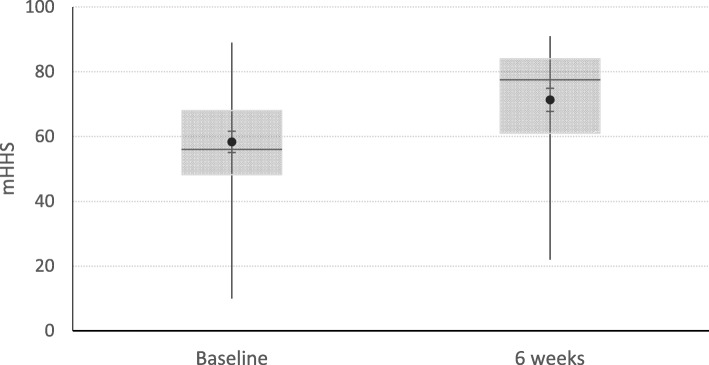
MHHS at baseline and 6 weeks. Legend: Box and whisker plot showing the distribution of mHHS scores at baseline and 6 weeks. mHHS: modified Harris Hip Score,  mean,  median,  high-low line

Table 2 and Fig. 2 show the primary outcome scores. At baseline the mean mHHS was 58.47 (SD 14.31). At the 6-week follow up the mean mHHS improved to 71.30 (SD 16.46), a change of 12.83 (*p* < 0.01). This was greater than the minimal clinically important difference (MCID) of 10 for clinical improvement at 6 weeks.

**Table 2:** Primary outcome data - mHHS scores at baseline and 6 weeks

	Number (%)	Mean mHHS	SD	95% CI
Baseline	87 (100%)	58.47	14.82	55.31–61.31
6 weeks	82 (94%)	71.30	16.46	67.68–74.92

Legend: *mHH*S Modified Harris Hip Score, *SD* Standard Deviation, *CI* Confidence Intervals

### Adverse events

Table 3 shows the adverse events. There were no treatment-related significant adverse events. Post-treatment pain was classified as minor or moderate. Minor pain was injection site pain lasting less than 24 h requiring no treatment or mild analgesia (for example: Paracetamol/Acetaminophen). Moderate pain was any pain lasting more than 24 h or requiring stronger analgesia. Five patients reported minor pain lasting less than 24 h requiring no treatment or minimal analgesia. No patients reported any other adverse events.

**Table 3:** Adverse events

Adverse event	Number (%)
Minor pain	5 (6%)
Infection	0 (0%)

## Discussion

The findings of this study are in keeping with those of previous studies in other joints that intra-articular injection of HA in the setting of mild to moderate OA results in a clinically measurable improvement in pain and function persistent to a minimum of 6 weeks. The modified Harris Hip Score at 6 weeks improved from 58.47 (SD 14.31) at baseline to 71.30 (SD 16.46), a difference of 12.83 which was both statistically significant and sufficient to reach the minimally important clinical difference of 10.

In this cohort of patients, Durolane injection had an excellent safety profile with no significant complications during or immediately following injection. Adverse events were scarce, mild and short lived in this cohort out to the 6-week timepoint with only 5 patients reporting injection site pain lasting between 12 and 24 h. Furthermore, no subsequent notifications have been made by this cohort of patients since the study period concluded. No patients in this study reported any of the previously described adverse events associated with HA injection including bleeding, local or systemic allergic response, septic arthritis or so-called “pseudoseptic arthritis”. It is hypothesised that non-animal derived HA is associated with decreased rates of allergic response and pseudoseptic arthritis and our results support this [1, 18, 22]. The use of ultrasound guidance also reduces the negative outcomes of misplaced injection [8, 25].

Previous studies have demonstrated clinical improvement with use of intra-articular HA in hip OA and the results of this study support these previous results [7, 9, 10, 12, 14, 16, 19]. However, controversy still exists in the literature. Holen et al. in a recent systematic review found a benefit of intra-articular HA in joints outside of the knee (including the hip joint) but concluded that the benefit was not better than placebo [10]. Rutjes et al. and Jevsevar et al. similarly found a small but clinically irrelevant benefit in HA in the knee [21, 34]. However the findings of Xing et al. in a recent PRISMA-compliant meta-analysis appear to contradict these former reviews and the authors go on to criticise Rutjes conclusions in particular due to the inclusion of unpublished, unverifiable data [39]. It is important to note, however, that many of these systematic reviews do not differentiate between different types of HA in clinical use and thus may be at risk of combining data from studies using interventions that have quite distinct molecular structure and efficacy.

Hyaluronic acid is a major component of synovial fluid which provides lubrication and shock absorption in joints. In OA there has been shown to be a decrease in HA molecular weight (MW) and concentration in the synovial fluid as the disease progresses, leading to a reduction in viscoelastic properties [7]. There is potential for symptomatic improvement by supplementing HA with better viscoelastic properties. The OARSI guidelines for the management of OA in 2014 gave HA an *uncertain* recommendation with insufficient evidence to make a recommendation whilst the Australian Royal Australian College of GPs (2018) guidelines recommended against their use largely based on minimal evidence from the knee & insufficient evidence at the hip [8, 10]. Several studies have, however, demonstrated significant superiority of high molecular weight and highly cross linked HA compared with low molecular weight and minimally cross linked HA [2, 21, 24]. Indeed, the recent consensus statement of the Arthroscopy Association of Canada regarding injectable therapies in knee OA reviews the subgroup analyses of systematic reviews and meta-analyses and concludes that high molecular weight and highly cross-linked HA preparations show both a statistically and clinically significant reduction in pain [4]. This further strengthens the case for more robust studies to examine the safety and effectiveness of the theoretically optimal HA preparations.

### The strengths of this study

This study has confirmed the short-term benefit and safety of a single Durolane injection in hip OA, providing data to inform a randomised controlled trial to further evaluate this treatment compared with placebo. Based on the results of previous studies in knee OA, it was expected that the first time point for improvement in symptoms would be at 6 weeks but further research will be required to determine longer term efficacy [24].

### The limitations of this study

The data from this study are gathered from a cohort of patients in a single geographical location with limited ethnic diversity and from a higher socioeconomic background given the nature and location of the treatment (private clinic setting). Whilst there was a range of ages and gender balance which could be seen to be representative of a normal population in this region, it is not possible to say with certainty if these findings are generalisable to populations beyond this cohort. This was an open-label study in which patients and assessors were not blinded to the treatment provided and we acknowledge the potential for placebo effect. Participants were advised to continue their current exercise regimen and take analgesia as required and these were not specifically documented as part of the study. The study does however, confirm the safety of Durolane in hip OA and infers potential for clinical efficacy. Further randomised controlled trials are required to confirm efficacy compared to placebo or control groups based on data from this study.

## Conclusion

The results of this study show that a single injection of HA (Durolane) in the setting of hip joint OA provides pilot data relating to safety and efficacy in this series of 87 patients. The modified Harris Hip Score at 6 weeks improved from 58.47 (SD 14.31) at baseline to 71.30 (SD 16.46), a difference of 12.83 (*p* < 0.01) which was both statistically significant and sufficient to reach the minimally important clinical difference of 10. However, this is preliminary data that requires confirmation in randomised controlled trials to further evaluate this treatment compared with placebo.

## Data Availability

The datasets used and/or analysed during the current study are available from the corresponding author on reasonable request.

## References

[1] Altman R, Manjoo A, Fierlinger A, Niazi F, Nicholls M. The mechanism of action for hyaluronic acid treatment in the osteoarthritic knee: a systematic review. BMC Musculoskel Disord. 2015;16(1). 10.1186/s12891-015-0775-z. 10.1186/s12891-015-0775-z PMC4621876 26503103

[2] Altman RD, Bedi A, Karlsson J, Sancheti P, Schemitsch E (2016). Product differences in intra-articular hyaluronic acids for osteoarthritis of the knee. Am J Sports Med.

[3] AOANJRR. Australian Orthopaedic Association National Joint Replacement Registry 2019.pdf. 2019. Accessed 22 Oct 2019.

[4] Kopka M, Sheehan B, Arthroscopy Association of Canada (2019). Arthroscopy association of Canada position statement on intra-articular injections for knee osteoarthritis. Orthop J Sports Med.

[5] Bannuru RR, Osani MC, Vaysbrot EE, Arden NK, Bennell K, Bierma-Zeinstra SMA, Kraus VB, Lohmander LS, Abbott JH, Bhandari M, Blanco FJ, Espinosa R, Haugen IK, Lin J, Mandl LA, Moilanen E, Nakamura N, Snyder-Mackler L, Trojian T, Underwood M, McAlindon TE (2019). OARSI guidelines for the non-surgical management of knee, hip, and polyarticular osteoarthritis. Osteoarthr Cartil.

[6] Basedow M, Williams H, Shanahan EM, Runciman WB, Esterman A (2015). Australian GP management of osteoarthritis following the release of the RACGP guideline for the non-surgical management of hip and knee osteoarthritis. BMC Res Notes.

[7] Battaglia M, Guaraldi F, Vannini F, Rossi G, Timoncini A, Buda R, Giannini S (2013). Efficacy of ultrasound-guided intra-articular injections of platelet-rich plasma versus hyaluronic acid for hip osteoarthritis. Orthopedics..

[8] Bossert M, Boublil D, Parisaux J-M, Bozgan A-M, Richelme E, Conrozier T (2016). Imaging guidance improves the results of Viscosupplementation with HANOX-M-XL in patients with ankle osteoarthritis: results of a clinical survey in 50 patients treated in daily practice. Clin Med Insights Arthritis Musculoskelet Disord.

[9] Clementi D, D’Ambrosi R, Bertocco P, Bucci MS, Cardile C, Ragni P, Giaffreda G, Ragone V (2018). Efficacy of a single intra-articular injection of ultra-high molecular weight hyaluronic acid for hip osteoarthritis: a randomized controlled study. Eur J Orthop Surg Traumatol.

[10] Colen S, Haverkamp D, Mulier M, van den Bekerom MPJ (2012). Hyaluronic acid for the treatment of osteoarthritis in all joints except the knee: what is the current evidence?. BioDrugs..

[11] Cross M, Smith E, Hoy D, Nolte S, Ackerman I, Fransen M, Bridgett L, Williams S, Guillemin F, Hill CL, Laslett LL, Jones G, Cicuttini F, Osborne R, Vos T, Buchbinder R, Woolf A, March L (2014). The global burden of hip and knee osteoarthritis: estimates from the global burden of disease 2010 study. Ann Rheum Dis.

[12] Dallari D, Stagni C, Rani N, Sabbioni G, Pelotti P, Torricelli P, Tschon M, Giavaresi G (2016). Ultrasound-guided injection of platelet-rich plasma and hyaluronic acid, separately and in combination, for hip osteoarthritis: a randomized controlled study. Am J Sports Med.

[13] Edwards PK, Queen RM, Butler RJ, Bolognesi MP, Lowry BC (2016). Are range of motion measurements needed when calculating the Harris hip score?. J Arthroplast.

[14] Eymard F, Maillet B, Lellouche H (2017). Predictors of response to viscosupplementation in patients with hip osteoarthritis: results of a prospective, observational, multicentre, open-label, pilot study. BMC Musculoskelet Disord.

[15] Fast facts. Arthritis Australia. https://arthritisaustralia.com.au/what-is-arthritis/fastfacts/. Accessed 1 Feb 2019.

[16] Ferrero G, Sconfienza LM, Fiz F, Fabbro E, Corazza A, Dettore D, Orlandi D, Castellazzo C, Tornago S, Serafini G (2018). Effect of intra-articular injection of intermediate-weight hyaluronic acid on hip and knee cartilage: in-vivo evaluation using T2 mapping. Eur Radiol.

[17] Fransen M, McConnell S, Harmer AR, der Esch MV, Simic M, Bennell KL (2015). Exercise for osteoarthritis of the knee: a Cochrane systematic review. Br J Sports Med.

[18] Goldberg VM, Coutts RD (2004). Pseudoseptic reactions to Hylan Viscosupplementation: diagnosis and treatment. Clin Orthop Relat Res.

[19] Greenberg DD, Stoker A, Kane S, Cockrell M, Cook JL (2006). Biochemical effects of two different hyaluronic acid products in a co-culture model of osteoarthritis. Osteoarthr Cartil.

[20] Januel J-M, Chen G, Ruffieux C, Quan H, Douketis JD, Crowther MA, Colin C, Ghali WA, Burnand B, IMECCHI Group (2012). Symptomatic in-hospital deep vein thrombosis and pulmonary embolism following hip and knee arthroplasty among patients receiving recommended prophylaxis: a systematic review. JAMA..

[21] Jevsevar D, Donnelly P, Brown GA, Cummins DS (2015). Viscosupplementation for osteoarthritis of the knee: a systematic review of the evidence. J Bone Joint Surg Am.

[22] Kirchner M, Marshall D (2006). A double-blind randomized controlled trial comparing alternate forms of high molecular weight hyaluronan for the treatment of osteoarthritis of the knee. Osteoarthr Cartil.

[23] Kyu HH, Abate D, Abate KH, Abay SM, Abbafati C, Abbasi N, et al. Global, regional, and national disability-adjusted life-years (DALYs) for 359 diseases and injuries and healthy life expectancy (HALE) for 195 countries and territories, 1990–2017: a systematic analysis for the global burden of disease study 2017. Lancet. 2018;392(10159):1859–922. 10.1016/S0140-6736(18)32335-3. 10.1016/S0140-6736(18)32335-3 PMC6252083 30415748

[24] Leighton R, Fitzpatrick J, Smith H, Crandall D, Flannery CR, Conrozier T (2018). Systematic clinical evidence review of NASHA (Durolane hyaluronic acid) for the treatment of knee osteoarthritis. Open Access Rheumatol.

[25] Lundstrom ZT, Sytsma TT, Greenlund LS (2020). Rethinking Viscosupplementation: ultrasound- versus landmark-guided injection for knee osteoarthritis. J Ultrasound Med.

[26] Mahomed NN, Arndt DC, McGrory BJ, Harris WH (2001). The Harris hip score: comparison of patient self-report with surgeon assessment. J Arthroplast.

[27] Maneiro E, de Andres MC, Fernández-Sueiro JL, Galdo F, Blanco FJ (2004). The biological action of hyaluronan on human osteoartritic articular chondrocytes: the importance of molecular weight. Clin Exp Rheumatol.

[28] McAlindon TE, LaValley MP, Harvey WF (2017). Effect of intra-articular triamcinolone vs saline on knee cartilage volume and pain in patients with knee osteoarthritis. JAMA..

[29] Moreland LW (2003). Intra-articular hyaluronan (hyaluronic acid) and hylans for the treatment of osteoarthritis: mechanisms of action. Arthritis Res Ther..

[30] Morrey BF (1997). Difficult complications after hip joint replacement. Dislocation. Clin Orthop Relat Res.

[31] Ottaviani RA, Wooley P, Song Z, Markel DC (2007). Inflammatory and immunological responses to hyaluronan preparations. Study of a murine biocompatibility model. J Bone Joint Surg Am.

[32] de la Peña E, Sala S, Rovira JC, Schmidt RF, Belmonte C (2002). Elastoviscous substances with analgesic effects on joint pain reduce stretch-activated ion channel activity in vitro. Pain..

[33] Plaas A, Li J, Riesco J, Das R, Sandy JD, Harrison A (2011). Intraarticular injection of hyaluronan prevents cartilage erosion, periarticular fibrosis and mechanical allodynia and normalizes stance time in murine knee osteoarthritis. Arthritis Res Ther.

[34] Rutjes AWS, Jüni P, da Costa BR, Trelle S, Nüesch E, Reichenbach S (2012). Viscosupplementation for osteoarthritis of the knee: a systematic review and meta-analysis. Ann Intern Med.

[35] Tamer TM (2013). Hyaluronan and synovial joint: function, distribution and healing. Interdiscip Toxicol.

[36] The Royal Australian College of General Practitioners. guideline-for-the-management-of-knee-and-hip-oa-2nd-edition.pdf. 2018. https://www.racgp.org.au/download/Documents/Guidelines/Musculoskeletal/guideline-for-the-management-of-knee-and-hip-oa-2nd-edition.pdf. Accessed 17 Jan 2019.

[37] Vina ER, Kwoh CK (2018). Epidemiology of osteoarthritis: literature update. Curr Opin Rheumatol.

[38] WHO | Disease burden and mortality estimates. WHO. http://www.who.int/healthinfo/global_burden_disease/estimates/en/. Accessed 19 Jan 2020.

[39] Xing D, Wang B, Liu Q, Ke Y, Xu Y, Li Z, et al. Intra-articular hyaluronic acid in treating knee osteoarthritis: a PRISMA-compliant systematic review of overlapping Meta-analysis. Sci Rep. 2016;6(1). 10.1038/srep32790. 10.1038/srep32790 PMC5018721 27616273

[40] Zhang W, Ouyang H, Dass CR, Xu J (2016). Current research on pharmacologic and regenerative therapies for osteoarthritis. Bone Res.

[41] Zhang Y, Jordan JM (2010). Epidemiology of osteoarthritis. Clin Geriatr Med.

